# Cardiometabolic dysfunction burden and mortality outcomes in metabolic dysfunction-associated steatotic liver disease

**DOI:** 10.1371/journal.pone.0327772

**Published:** 2025-07-03

**Authors:** Ying Wen, Yu Min, Yi Lei, Zhigong Wei

**Affiliations:** 1 Department of Biotherapy, Cancer Center, West China Hospital, Sichuan University, Chengdu, Sichuan, People's Republic of China; 2 Department of Endocrinology and Metabolism, Affiliated Hospital of Southwest Medical University, Luzhou, Sichuan, People's Republic of China; Instituto Nacional de Ciencias Medicas y Nutricion Salvador Zubiran, MEXICO

## Abstract

**Background/Objectives:**

The term metabolic dysfunction-associated steatotic liver disease (MASLD) reflects the key role of cardiometabolic dysfunction in steatotic liver disease. We aim to assess the association between cardiometabolic dysfunction burden and mortality outcomes in MASLD.

**Methods:**

Participants with MASLD were selected from the National Health and Nutrition Examination Survey (NHANES) database between 1999 and 2018. The mortality outcomes of participants with different numbers of cardiometabolic risk factors were compared by using Kaplan-Meier curves and Cox regression analysis.

**Results:**

This study included 9,017 participants with MASLD (4,613 men and 4,404 women, median age 49.0). With a median 115-month follow-up, 1,447 all-cause deaths and 407 cardiovascular-specific deaths were observed. Multivariate regression analysis showed that participants with five cardiometabolic risk factors had significantly increased all-cause mortality risk compared to those with one risk factor (adjusted hazard ratio [aHR] = 3.57, 95% confidence interval [CI]: 2.04–6.24, *P* < 0.001). Similarly, the cardiovascular mortality risk was markedly higher for participants with five risk factors (aHR = 7.72, 95% CI: 1.89–31.53, *P* = 0.004). Among participants with the same number of cardiometabolic risk factors, those with blood glucose or blood pressure abnormalities showed the lowest survival rates than other subgroups. Besides, participants with younger ages were more vulnerable to the harmful prognostic effects of cardiometabolic dysfunction burden on the mortality risks.

**Conclusions:**

The MASLD population with high cardiometabolic dysfunction burdens exhibits increased mortality risk. Assessing cardiometabolic dysfunction, particularly abnormalities in blood glucose and blood pressure, is crucial for effective management in this population.

## 1. Introduction

Following the recommendations of a recent multi-society expert consensus, the term metabolic dysfunction-associated steatotic liver disease (MASLD) has been introduced to replace nonalcoholic fatty liver disease (NAFLD), to emphasize the central role of systemic metabolic dysfunction in the pathogenesis of hepatic steatosis, while also avoiding the negative labeling and stigma associated with the term “nonalcoholic” [1]. Compared to NAFLD, MASLD incorporates a broader etiological spectrum of steatotic liver disease (SLD) and explicitly integrates cardiometabolic abnormalities as a core component of disease definition. This updated terminology is anticipated to better reflect disease biology, improve epidemiological tracking, and enhance opportunities for biomarker identification and therapeutic innovation [1,2]. As a result, MASLD has garnered growing attention for its potential to reshape clinical risk stratification, define novel therapeutic targets, and deepen our understanding of extrahepatic complications compared to NAFLD [3]. However, prognostic heterogeneity remains a challenge within the MASLD population, and current knowledge regarding the factors influencing long-term outcomes is still less explored [4–6]. Therefore, identifying additional prognostic indicators, particularly those reflecting the burden of cardiometabolic dysfunction, may contribute to a more refined risk assessment and comprehensive clinical management in individuals with MASLD [4].

Notably, regarding the key role of systemic metabolic disorders in the occurrence and progress of SLD, one study from the UK Biobank revealed that SLD patients with metabolic abnormalities experienced a significantly higher number of cardiovascular events as well as mortality risk than those experienced by SLD and non-SLD subjects [2]. Similarly, the consistent findings were also determined in another large-scale cohort study from South Korea [7]. Therefore, cardiometabolic dysfunction was an independent risk factor for adverse events and survival, regardless of the presence of SLD. Although SLD (excluding other etiological factors) population with one or more cardiometabolic risk factors can be diagnosed as MASLD according to the Delphi consensus process, different types and numbers of cardiometabolic risk factors might be involved in the diagnosis process, resulting in the heterogeneity of cardiometabolic dysfunction burden in individuals. Nevertheless, there remains a lack of robust evidence on the association between the number of cardiometabolic risk factors and the long-term prognosis of the MASLD population [4]. Addressing this research gap would help to enhance our understanding of the comprehensive health implications of MASLD and develop more cost-effective management strategies for affected individuals.

Thereby, we aim to explore the prognostic effects of cardiometabolic dysfunction burden, measured by the number of cardiometabolic risk factors, in the mortality outcomes of MASLD based on a representative population-based cohort. Additionally, we also aim to evaluate the most harmful risk cardiometabolic risk factors in the MASLD population. This study might bring additional knowledge to refine the clinical management of the MASLD population.

## 2. Materials and methods

### 2.1 Data source and study population

Data in this study were collected from the National Health and Nutrition Examination Survey (NHANES) database, one representative large-scale ongoing database, between 1999 and 2018. The NHANES program systematically gathers nationally representative health-related data on the non-institutionalized US population, utilizing a stratified, multistage probability sampling design [8]. Specific descriptions of the NHANES database can be found elsewhere [9–11]. The NHANES 1999–2018 data utilized in this study were approved by the National Center for Health Statistics Ethics Review Board (NCHS ERB) under the following protocol numbers: Protocol #98−12 (1999–2004), Protocol #2005−06 (2005–2010), Protocol #2011−17 (2011–October 26, 2017), and Protocol #2018−01 (from October 26, 2017 onward) (https://www.cdc.gov/nchs/nhanes/about/erb.html). Written informed consent to participate in the NHANES program was obtained from all participants. This study was conducted in compliance with local legislation and the principles expressed in the Declaration of Helsinki. As a secondary analysis of publicly available and de-identified data, this study was exempt from additional ethical review by our institutional ERB of West China Hospital.

**Fig 1:**
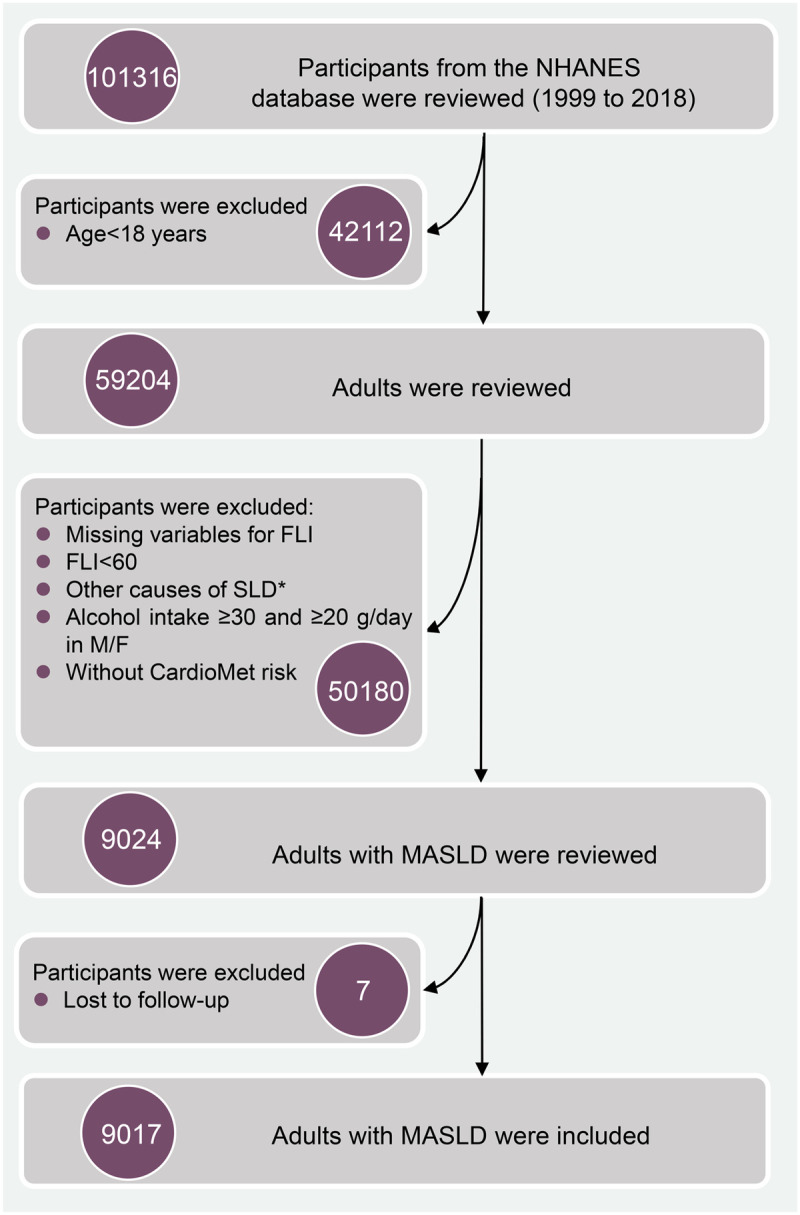
The selection process of participants with MASLD. *Other potential causes of SLD include viral hepatitis, autoimmune liver disease, genetic liver diseases, drug- or medication-induced liver disease, and alcohol-related liver disease. MASLD: metabolic dysfunction-associated steatotic liver disease; SLD: steatotic liver disease; FLI: fatty liver index.

Among ten cycles of interviews, a total of 101,316 participants were initially screened. After excluding non-adult participants, 59,204 participants remained. Next, participants with missing data on cardiometabolic factors, including triglycerides (TG), fasting blood glucose (FBG), and anthropometric measurements, were excluded. Participants presenting clinical features of the fatty liver index (FLI) < 60, with other causes of SLD, having moderate to heavy daily alcohol intake (≥ 30 g/day for men and ≥ 20 g/day for women), or lost to follow-up were further excluded. Finally, 9,017 participants with MASLD were included in this study (Fig 1).

The publicly available and de-identified NHANES datasets were accessed for research purposes on 12 Dec 2024. The authors did not have access to any information that could identify individual participants during or after data collection. This study is reported followed the Strengthening the Reporting of Observational Studies in Epidemiology (STROBE) [12].

### 2.2 Diagnosis of MASLD

In this large-scale epidemiological study, hepatic steatosis was assessed by using the FLI, a cost-effective screening tool for detecting SLD in population-based studies [13–15], with good sensitivity and specificity. The equation is listed below:

FLI = (e^0.953 × Ln (triglycerides) + 0.139 × body mass index + 0.718 × Ln (gamma-glutamyl transferase) + 0.053 × waist circumference − 15.745^)/ (1 + e^0.953 × Ln (triglycerides) + 0.139 × body mass index + 0.718 × Ln (gamma-glutamyl transferase) + 0.053 × waist circumference − 15.745^) × 100 [16].

According to the report from the original equation designers, participants with FLI < 60 were considered to have a low probability of hepatic steatosis, while those with FLI ≥ 60 were considered to have a high probability of hepatic steatosis [13,16]. Therefore, participants with FLI ≥ 60 were diagnosed with SLD.

The pure MASLD was diagnosed according to the latest literature [1]. First, SLD participants with viral hepatitis, autoimmune liver disease, genetic liver diseases, drug- or medication-induced liver disease, alcohol-related liver disease, or alcohol intake of ≥30 g/day for men and ≥20 g/day for women were excluded from the study population. Then, the MASLD was defined as the presence of steatotic liver disease (SLD) accompanied by at least one of five cardiometabolic risk factors [1]: (1) elevated waist circumference (WC) or body mass index (BMI); (2) elevated blood pressure or use of antihypertensive medications; (3) elevated triglycerides or use of lipid-lowering agents; (4) reduced HDL-cholesterol; and (5) elevated blood glucose, increased hemoglobin A1c, or use of hypoglycemic agents for diabetes mellitus (DM). The detailed diagnostic criteria for MASLD are provided in S1 Table.

### 2.3 Cardiometabolic dysfunction burden assessment

Participants with MASLD were classified into five subgroups based on the number of cardiometabolic risk factors. Participants with more cardiometabolic risk factors indicated a greater metabolic burden. Therefore, they were classified into five subgroups: participants with only one cardiometabolic risk factor, participants with two cardiometabolic risk factors, participants with three cardiometabolic risk factors, participants with four cardiometabolic risk factors, and participants with five cardiometabolic risk factors. The subgroup with only one cardiometabolic risk factor was set as the reference.

### 2.4 Demographic feature and covariates assessment

Demographic features of participants with MASLD were collected from the NHANES database at the same time. First, the socioeconomic features, including sex (male and female), age at the interview, race (Hispanic, non-Hispanic White, non-Hispanic Black, and other races), marital status at interview (not married, married, and living with partner), educational level at interview (≤ high school, college, and> college), and family poverty income ratio (PIR, < 1.3, 1.3–3.5, or >3.5) were recorded. Additionally, personal habits, including smoking status (never smoking, ever smoking, and currently smoking), and alcohol use (never drinking, ever drinking, and currently drinking) were collected. Meanwhile, the history of comorbidities, including DM, cancer, chronic kidney disease (CKD), and cardiovascular disease (CVD), was collected. Moreover, physical and laboratory examinations, including height, BMI, WC, daily energy intake (calculated by using the average kilocalorie of two 24-hour dietary recall interviews), total bilirubin (TBil), triglyceride (TG), fasting blood glucose (FBG), Platelet (PLT), total cholesterol (TC), alanine aminotransferase (ALT), and aspartate aminotransferase (AST) were also collected.

### 2.5 Follow-up and mortality assessments

The follow-up period of the study population was calculated from the date of the initial interview to either the date of death or December 31, 2019 [17]. The primary outcome of this study was the all-cause and cardiovascular mortality of participants with MASLD. Mortality data for the follow-up population were obtained from the NCHS using the National Death Index (NDI). The all-cause mortality was defined as death due to any cause, whereas cardiovascular mortality was defined by the ICD-10 (International Statistical Classification of Diseases, 10th revision) codes.

### 2.6 Statistical analysis

According to the official guideline for analytic procedures using the NHANES database (https://wwwn.cdc.gov/nchs/nhanes/tutorials), all analyses in this study incorporated sample weights, clustering, and stratification to estimate appropriate variance and make sure the national representation of the U.S. population with MASLD.

The continuous variables in this study were non-normally distributed. Therefore, they were presented as median (interquartile range, Q_1_, Q_3_). Categorical variables were presented as numbers (percentages, %). Continuous variables between survivors and non-survivors were compared using the Mann-Whitney U test, while categorical variables were compared using the Chi-squared test with Rao & Scott’s second-order correction.

Multivariate Cox regression analyses with the Wald test were used to estimate the association between varying cardiometabolic dysfunction burdens (calculated as the number of cardiometabolic risk factors) and all-cause and cardiovascular mortality among the MASLD population. Potential confounders were selected based on their associations with the outcomes of interest mentioned in previous works [18,19]. Specifically, Model 1 was not adjusted for covariates. Model 2 was briefly adjusted for age, sex, and race. In Model 3, age, sex, race, marital status, educational level, PIR, smoking status, alcohol use, cancer, CKD, CVD, energy intake, and serum levels of TC, ALT, AST, and TBil were fully adjusted. Results were displayed as hazard ratio (HR) with 95% confidence interval (95%CI). Kaplan-Meier (KM) curves were analyzed to show the censored data and different survival probabilities of participants with MASLD at different burdens of cardiometabolic dysfunction. Additionally, we evaluated the survival rates of obese participants with different cardiometabolic risk factors at the same cardiometabolic dysfunction burden. To further evaluate the impact of cardiometabolic control intensity on the prognosis of the MASLD population, we conducted subgroup analyses among MASLD participants who concurrently had all three major cardiometabolic risk factors (Diabetes, hypertension, and dyslipidemia) and received corresponding glucose-lowering, antihypertensive-, and lipid-lowering treatments. In this high-risk subgroup, we examined the association between the degree of target control (full, partial, or none) and all-cause mortality. Full target control was defined as achieving blood pressure <130/85 mmHg, LDL-c < 1.8 mmol/L, and HbA1c < 7.0%. Additionally, we assessed the prognostic differences in the treated MASLD population based on the number of cardiometabolic risk factors. Missing data were imputed using the multiple imputation method [20,21].

We conducted a series of sensitive analyses to check the robustness of our findings. First, to evaluate the potential impact of liver disease severity on the association between cardiometabolic dysfunction burden and mortality among individuals with MASLD, we conducted stratified sensitivity analyses based on baseline Fibrosis-4 (FIB-4) index levels, a widely used noninvasive surrogate marker for liver fibrosis. FIB-4 scores were calculated using the following equation:

FIB-4 = Age (year) × AST [U/L]/ (PLT [10^9^/L] ×ALT [U/L]^1/2^) [22].

Participants were stratified into low (< 1.3), intermediate -to-high (≥1.3) advanced fibrosis-risk categories to approximate liver disease severity in the absence of biopsy or imaging data [23–25]. This stratification was used to test whether the observed mortality associations remained consistent across different advanced fibrosis risk levels.

Second, we excluded participants who died within two years after the interview to reduce potential reverse causality between exposure and outcome. Second, we included participants with FLI ≥ 30 [15] for the diagnosis of SLD to check the association in a more generalizable population. Lastly, we evaluated the main findings in a strict population from 1999 to 2008 to test the potential impact of time variation on the survival of participants with MASLD.

Data were organized using Microsoft Excel (Microsoft Corp, LA, USA) and STATA 16.0 (Stata Corp, College Station, TX, USA). All statistical analyses were conducted using R 4.3.2 (R Foundation, Vienna, Austria). Two-tail *P*-value < 0.05 was considered statistically significant.

## 3. Results

### 3.1 Demographic characteristics of the participants with MASLD

Of the 101,316 participants, 9,017 participants (4,613 men and 4,404 women, median age 49.00 years) were diagnosed with MASLD and ultimately included in this study. The non-Hispanic White population accounted for a majority of the study population (3,898, 68.7%), and over one-third of the study population were not married (3,495, 34.1%). Approximately half (n = 4,145) of the participants with MASLD were ever or currently smoking. As for comorbidities, 9.3% (n = 997) of participants with MASLD had a history of CVD, 2.8% (n = 322) had a history of CKD, 9.7% (n = 849) had a history of cancer, and 15.2% (n = 1,630) had a history of diabetes mellitus. Among the MASLD participants, 5.8% (n = 481) had one cardiometabolic risk factor, 17.1% (n = 1,494) had two, 26.6% (n = 2,362) had three, 26.7% (n = 2,400) had four, and 23.8% (n = 2,280) had five cardiometabolic risk factors at baseline. With a median follow-up of 115 months, there were 1,447 all-cause deaths and 407 cardiovascular-specific deaths. Non-survivors were more likely to be older, male, non-Hispanic White, with lower BMI, lower energy intake, higher numbers of cardiometabolic risk factors, lower socioeconomic status, and concurrent comorbidities. The number of cardiometabolic risk factors was positively associated with higher FLI in participants with MASLD (S1 Fig). The specific clinical characteristics of study participants are shown in Table 1.

**Table 1:** The demographic characteristics of the population with MASLD.

Variables	Total (n = 9,017)	Survivors (n = 7,570, 88.2%)	Non-survivors (n = 1,447, 11.8%)	P
AST, M (Q₁, Q₃)	23.00 (19.00,28.00)	23.00 (19.00,28.00)	23.00 (19.00,28.00)	0.293
ALT, M (Q₁, Q₃)	25.00 (18.00,33.00)	25.00 (19.00,34.00)	21.00 (17.00,28.00)	**<0.001**
TBil, M (Q₁, Q₃)	10.26 (8.55,13.68)	10.26 (8.55,13.68)	11.97 (8.60,15.39)	**<0.001**
BMI, M (Q₁, Q₃)	33.10 (30.00,37.30)	33.20 (30.15,37.40)	31.98 (29.02,36.10)	**<0.001**
FLI, M (Q₁, Q₃)	85.54 (73.63,94.62)	85.42 (73.56,94.59)	86.16 (75.22,94.82)	0.078
Energy intake, M (Q₁, Q₃)	2,008.64 (1,517.00,2,671.55)	2,056.93 (1,556.00,2,724.00)	1,764.33 (1,317.00,2,325.80)	**<0.001**
No. of Cardiometabolic risk factors, n (%)				**<0.001**
One risk factor	481 (5.8)	468 (6.3)	13 (1.6)	
Two risk factors	1,494 (17.1)	1,368 (18.1)	126 (8.6)	
Three risk factors	2,362 (26.6)	2,039 (27.2)	323 (22.8)	
Four risk factors	2,400 (26.7)	1,947 (26.2)	453 (30.1)	
Five risk factors	2,280 (23.8)	1,748 (22.2)	532 (36.9)	
Sex, n (%)				**0.015**
Male	4,613 (54.3)	3,763 (52.8)	850 (57.9)	
Female	4,404 (45.7)	3,807 (47.2)	597 (42.1)	
Race, n (%)				**<.001**
Hispanic	2,757 (16.0)	2,446 (17.0)	311 (8.0)	
Non-Hispanic White	3,898 (68.7)	3,068 (67.1)	830 (78.2)	
Non-Hispanic Black	1,862 (10.6)	1,588 (11.0)	274 (10.7)	
Others	500 (4.7)	468 (4.9)	32 (3.1)	
Education level, n (%)				**<.001**
≤High school	5,081 (46.6)	4,100 (44.8)	981 (60.1)	
College	2,471 (31.7)	2,175 (32.0)	296 (25.9)	
>College	1,465 (21.7)	1,295 (23.2)	170 (14.0)	
Marital status, n (%)				**0.014**
Not married	3,495 (34.1)	2,876 (32.8)	619 (37.7)	
Married/living with partner	5,522 (65.9)	4,694 (67.2)	828 (62.3)	
PIR, n (%)				**<.001**
<1.3	2,737 (20.8)	2,248 (20.2)	489 (25.7)	
1.3-3.5	3,984 (42.1)	3,285 (40.7)	699 (48.6)	
>3.5	2,296 (37.1)	2,037 (39.1)	259 (25.7)	
Smoking, n (%)				**<.001**
Never	4,872 (53.2)	4,314 (55.3)	558 (35.7)	
Now	1,582 (18.2)	1,334 (18.2)	248 (22.1)	
Ever	2,563 (28.6)	1,922 (26.5)	641 (42.2)	
Alcohol use, n (%)				**<.001**
Never	2,136 (19.7)	1,617 (18.1)	519 (35.1)	
Now	5,031 (63.1)	4,401 (65.0)	630 (46.8)	
Ever	1,850 (17.2)	1,552 (16.9)	298 (18.1)	
CVD, n (%)				**<.001**
No	8,020 (90.7)	6,992 (93.2)	1,028 (71.9)	
Yes	997 (9.3)	578 (6.8)	419 (28.1)	
CKD, n (%)				**<.001**
No	8,695 (97.2)	7,347 (97.6)	1,348 (94.4)	
Yes	322 (2.8)	223 (2.4)	99 (5.6)	
Cancer, n (%)				**<.001**
No	8,168 (90.3)	6,995 (91.7)	1,173 (79.9)	
Yes	849 (9.7)	575 (8.3)	274 (20.1)	
Diabetes mellitus, n (%)				**<.001**
No	7,387 (84.8)	6,400 (86.9)	987 (72.1)	
Yes	1,630 (15.2)	1,170 (13.1)	460 (21.9)	

Abbreviation: MASLD: metabolic dysfunction-associated steatotic liver disease; M: median; Q: quartile; TC: total cholesterol; ALT: glutamic-pyruvic transaminase; AST: aspartate transaminase; CKD: chronic kidney disease; CVD: cardiovascular disease; TBil: total bilirubin; DM: diabetes mellitus; BMI: body mass index; FLI: fatty liver index; PIR: poverty income ratio.

Note: Continuous variables were presented as median (interquartile range). The categorical variables were presented as numbers (weighted percentage, %). Continuous variables between survivors and non-survivors were compared with the Mann-Whitney U test, whereas categorical variables were compared by using the Chi-squared test with Rao & Scott’s second-order correction. Bold value means statistically significant (*P* < 0.05).

### 3.2 Associations between cardiometabolic dysfunction burden and prognosis of MASLD

**Fig 2:**
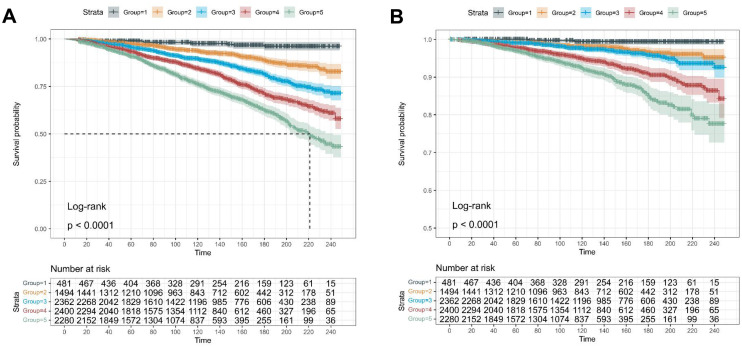
The Kaplan-Meier curves (Log-rank test) display the survival rate of MASLD participants with different numbers of cardiometabolic risk factors. (A) All-cause mortality; (B) Cardiovascular mortality.

The Kaplan-Meier analysis showed that a higher number of cardiometabolic risk factors in participants with MASLD was associated with worse all-cause and cardiovascular-specific survival (Fig 2A and 2B) (All Log-rank *P* < 0.0001). Among obese participants with one additional cardiometabolic risk factor, those with blood pressure or glucose abnormalities presented lower survival rates than the other subgroups (*P* < 0.0001) (S2 Fig).

Among obese participants with two additional cardiometabolic risk factors, those with blood pressure and glucose abnormalities presented the lowest survival rate compared to other subgroups (P < 0.0001) (S3 Fig). Moreover, obese participants concurrent with three cardiometabolic risk factors (blood pressure, glucose, and TG abnormalities) showed the worst survival probability compared to other subgroups (S4 Fig).

**Fig 3:**
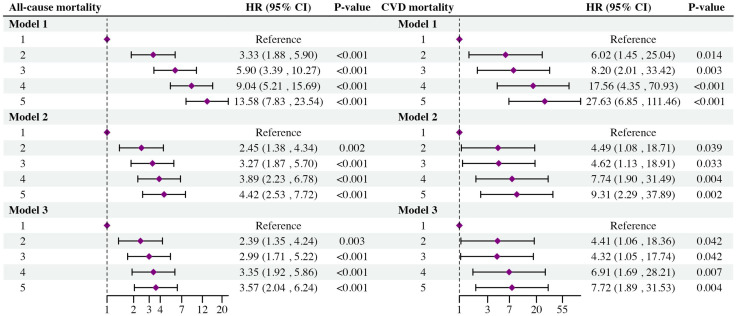
The Cox regression analysis models show the association between the number of cardiometabolic risk factors and the mortality outcomes of participants with MASLD (Wald test). Model 1: unadjusted model; Model 2: adjusted for age, sex, and race; Model 3: adjusted for age, sex, race, marital status, educational level, poverty income ratio, energy intakes, smoking status, alcohol use, CVD, CKD, cancer, AST, ALT, TBil, and TC. MASLD: metabolic dysfunction-associated steatotic liver disease; HR: hazard ratio; CI: confidence interval. *P* < 0.05 indicates statistical significance.

To explore the independent prognostic effect of cardiometabolic dysfunction burden on mortality outcomes of MASLD, three Cox regression models were conducted. In the unadjusted Cox regression analysis model, MASLD participants with five cardiometabolic risk factors had significantly increased all-cause (adjusted HR [aHR] = 13.58, 95% CI: 7.83–23.45, *P* < 0.001) and cardiovascular mortality risk (aHR = 27.63, 95% CI: 6.85–111.46, *P* < 0.001), compared to those with only one risk factor. Consistent findings were also observed in a fully adjusted Cox regression model (Participants with five cardiometabolic risk factors *vs.* participants with one cardiometabolic risk factor: All-cause mortality: aHR = 3.57, 95% CI: 2.04–6.24, *P* < 0.001; Cardiovascular mortality: aHR = 7.72, 95% CI: 1.89–31.53, *P* = 0.004) (Fig 3).

### 3.3 Subgroup and sensitive analysis

**Fig 4:**
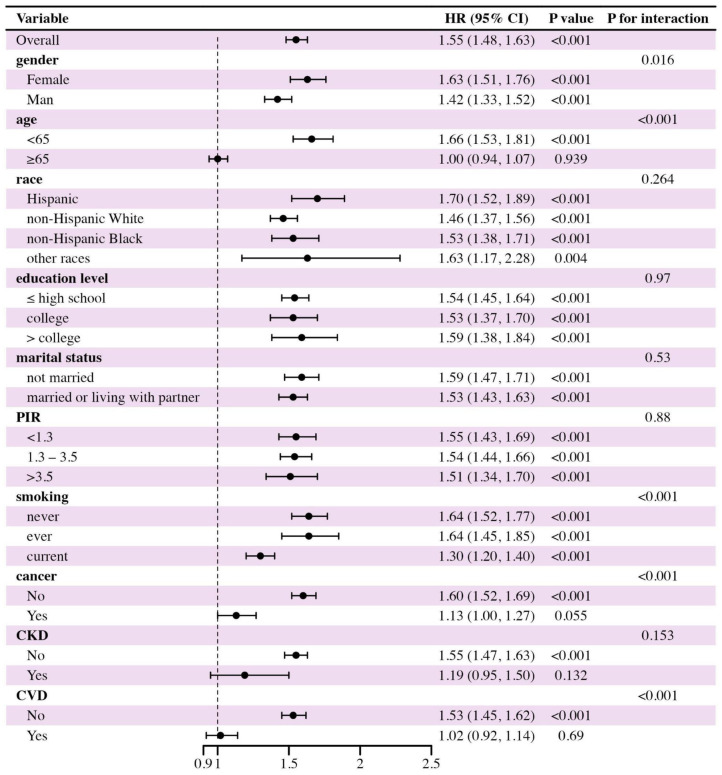
Subgroup analysis in the association between the number of cardiometabolic risk factors and the all-cause mortality of participants with MASLD. PIR: poverty income ratio; CKD: chronic kidney disease; CVD: cardiovascular disease. *P *< 0.05 indicates statistical significance.

**Fig 5:**
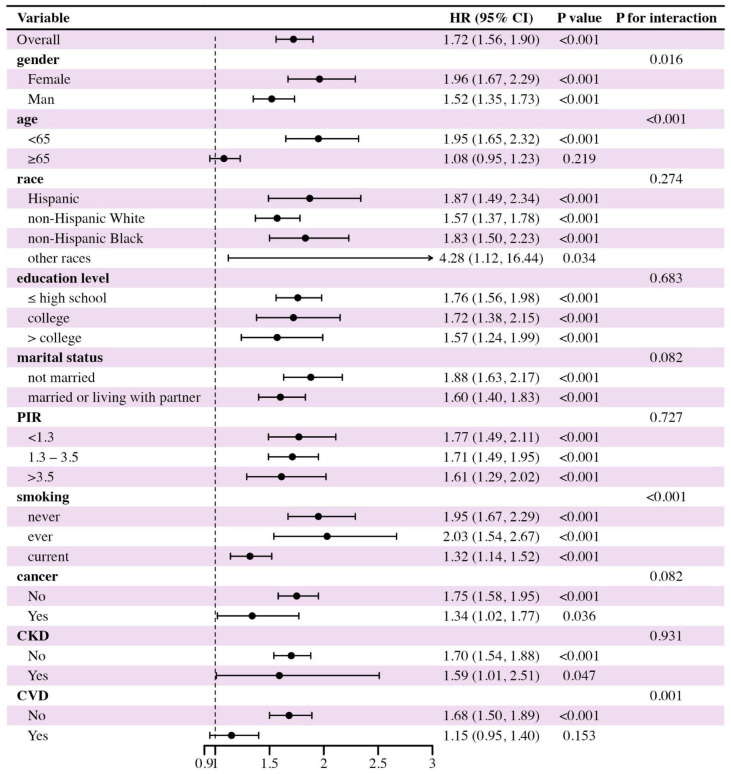
Subgroup analysis in the association between the number of cardiometabolic risk factors and the cardiovascular mortality of participants with MASLD. PIR: poverty income ratio; CKD: chronic kidney disease; CVD: cardiovascular disease. *P* < 0.05 indicates statistical significance.

We conducted subgroup analyses to further identify high-risk mortality populations. As shown in Fig 4 and Fig 5, MASLD participants who were younger, never or ever smokers, and without a history of CVD or cancer (for cardiovascular mortality only) were more vulnerable to the effects of cardiometabolic dysfunction burden on mortality risks.

Some interactions were observed between cardiometabolic dysfunction burden and other covariates, including sex, age, smoking status, and history of CVD. Besides, among treated MASLD patients with the same three major cardiometabolic risk factors (diabetes, hypertension, and dyslipidemia), those who failed to achieve any metabolic control had a nearly 2.2-fold higher risk of all-cause mortality compared to those who achieved full target control (aHR = 2.20, 95% CI: 1.26–3.86, *P* = 0.006; S2 Table). Furthermore, in this subgroup, patients with two or more treated cardiometabolic risk factors still exhibited significantly higher all-cause mortality than those with only one treated risk factor (aHR = 1.42, 95% CI: 1.03–1.95, *P* = 0.032; S3 Table). Notably, the association between cardiometabolic dysfunction burden and all-cause mortality remained significant regardless of FIB-4-defined fibrosis risk. Specifically, among MASLD participants with either low or intermediate-to-high risk of advanced fibrosis, a greater number of cardiometabolic risk factors was consistently associated with an increased risk of all-cause mortality (S4 Table). Also, the significantly positive association between cardiometabolic dysfunction burden and mortality outcomes of participants with MASLD remained robust after excluding participants who died within 24 months after the interview (S5 Table). Additionally, in a broader population (FLI ≥ 30), the independent prognostic effect of cardiometabolic dysfunction burden on participants with MASLD was observed (S6 Table). Moreover, in participants from the cycle between 1999 to 2008, a similar correlation between cardiometabolic dysfunction burden and mortality outcomes of participants with MASLD was observed (S7 Table).

## 4. Discussion

The findings of this large-scale, population-based cohort study demonstrate that the cardiometabolic dysfunction burden significantly impacts mortality outcomes in US adults with MASLD. Each additional cardiometabolic risk factor is associated with higher risks of both all-cause and cardiovascular mortality in adults with MASLD. Notably, among the five metabolic risk factors, abnormalities in blood glucose and blood pressure emerge as the predominant determinants adversely affecting survival outcomes of adults with MASLD. This study extends previous work by quantifying how metabolic risk factors collectively influence prognosis beyond their diagnostic role in MASLD, especially in the population with high cardiometabolic disorder burdens.

In accordance with the recent Delphi consensus, our study adopts the term MASLD, which emphasizes the etiological role of cardiometabolic dysfunction in steatotic liver disease [3,26]. While most existing evidence demonstrated a positive association between the presence of MASLD and elevated mortality risks, the impact of coexisting different types and quantities of cardiometabolic risk factors on the prognosis of adults with MASLD remained underexplored. Recent studies determined that systemic metabolic abnormalities, rather than SLD itself, were the key drivers of complications and mortality in MASLD [2,27]. Therefore, MASLD should not be treated as a homogeneous entity that individuals differ considerably in their underlying cardiometabolic profiles. Despite this, many studies have grouped MASLD as a whole, overlooking its internal heterogeneity [4]. Our study addresses this gap by identifying distinct survival outcomes across MASLD participants stratified by cardiometabolic dysfunction burden. Specifically, our data showed that fewer than 5.8% of the population had only one risk factor, while over half had three or more risk factors. Individuals with five cardiometabolic risk factors had a nearly 3.6-fold increased risk of all-cause mortality and a 7.72-fold increased risk of cardiovascular mortality compared to those in the low cardiometabolic dysfunction burden subgroup. These findings underscored the necessity of considering the internal cardiometabolic dysfunction burden when evaluating the prognostic impact of MASLD in various clinical settings.

Furthermore, overweight and obesity are highly prevalent in the population with MASLD, with a rate of 80% to 90% [28], consistent with the study population in the current work. Due to the high prevalence of obesity in the study population, it was challenging to disentangle the independent prognostic effect of BMI or waist circumference from other coexisting metabolic risk factors. This limitation highlights the need for future research with more refined phenotyping approaches, such as imaging- or biopsy-confirmed SLD, and larger, well-stratified cohorts to better clarify the individual contributions of anthropometric indicators to mortality risk in MASLD. Alternatively, we explored the mortality risk associated with different types of cardiometabolic risk factors in overweight or obese individuals with MASLD. Kaplan-Meier analyses revealed that blood pressure and FBG were the leading risk factors related to worse survival rates in MASLD participants with similar numbers of cardiometabolic risk factors. Insulin resistance and chronic systemic inflammation are key drivers of MASLD-associated cardiovascular events and hepatic complications [3,29]. The complex interplay between insulin resistance and chronic systemic inflammation could lead to lipid metabolic disorders, endoplasmic reticulum stress, oxidative stress, and mitochondrial dysfunction [30,31], further aggravating hepatocyte damage and the development of hepatic steatosis [32–35]. Our previous study with recent studies underscored the prognostic effects of insulin-resistant-related biomarkers and inflammation factors in the mortality outcomes of MASLD [36,37]. In addition, a recent comprehensive review demonstrated the bidirectional association between NAFLD and hypertension [38]. Notably, accumulating evidence supported that hypertension was an independent predictor for the onset as well as progress of NAFLD [39,40]. Meanwhile, the NAFLD condition would impair vascular compliance and increase the risk of arteriosclerosis and hypertension [41–43]. In participants with the new term MASLD, we determined the similar adverse effects of blood pressure abnormality in the mortality outcomes in this population. Our preliminary findings contributed to understanding the varied impact of five cardiometabolic risk factors on the clinical outcomes of MASLD. Further studies are warranted to validate our results, assisting clinicians in making tailored treatment strategies for the high-risk MASLD subpopulation.

Subgroup analysis revealed significant age differences in the impact of cardiometabolic dysfunction burden on mortality outcomes of MASLD, with younger individuals being more vulnerable to cardiometabolic dysfunction-associated mortality than older individuals. Previous works also indicated that the younger population is more susceptible to prognostic predictors in metabolic syndrome and MASLD [36,44–46]. Notably, Yu et al. found that the mortality risk was more pronounced in younger US individuals with a greater number of cardiometabolic dysfunction risk factors. In contrast, older adults with all five cardiometabolic dysfunction risk factors also had an increased mortality risk [46]. Thus, younger MASLD patients should receive more active surveillance for cardiometabolic variations. Besides, among patients with comparable baseline cardiometabolic risk factors, those who failed to achieve any metabolic control through treatment showed significantly higher mortality risks. This finding suggested that therapeutic responsiveness, defined by the degree of target achievement, might meaningfully modify the disease trajectory and improve prognosis. Furthermore, we found that the number of cardiometabolic risk factors remained a pivotal predictor of mortality, even among patients who were all under treatment. Specifically, individuals with three treated risk factors still experienced significantly higher mortality compared to those with only one, highlighting that the cumulative burden of metabolic dysfunction may exert a stronger prognostic influence than treatment efficacy alone. This observation suggested a residual risk hierarchy in which the absolute burden of metabolic dysregulation may outweigh isolated treatment success. Therefore, future risk stratification strategies in MASLD should incorporate not only the number of metabolic risk factors but also the duration and effectiveness of therapeutic interventions to better reflect the prognosis for this population.

In addition, stratified analysis by fibrosis severity using the FIB-4 index showed that the association between cardiometabolic dysfunction burden and all-cause mortality remained robust across both low and intermediate-to-high-risk groups for advanced fibrosis. This finding indicates that the metabolic dysfunction burden exerts a prognostic impact independent of underlying fibrosis severity. Nevertheless, given the limited number of participants classified into the high-risk FIB-4 category in our study, we combined the intermediate- and high-risk groups for analytic purposes. This limitation underscores the need for larger prospective studies with greater statistical power to explore more granular stratification by fibrosis stage. Furthermore, future studies employing more precise diagnostic modalities for liver fibrosis, such as transient elastography or liver biopsy, may help to validate and refine the prognostic implications of cardiometabolic dysfunction burden in MASLD across different fibrosis severities.

### Strength and limitation

This study has several notable strengths. First, leveraging a nationally representative cohort of over 100,000 U.S. adults with extended median follow-up (115 months), our findings minimize selection bias while reflecting real-world MASLD epidemiology. Second, we systematically quantified the prognostic gradient of cardiometabolic dysfunction burden, an underexplored dimension of MASLD heterogeneity, through multivariable-adjusted models that accounted for competing risks. Third, the stratification of treatment response patterns provides novel insights into the interplay between cardiometabolic control efficacy and residual risk persistence. Finally, the comprehensive sensitivity analyses and subgroup-specific mortality risk profiling proved the robustness of primary findings, which might collectively reduce potential confounding from reverse causality and cohort effects.

However, some limitations should be acknowledged. First, the diagnosis of steatotic liver disease (SLD) in this study was based on an FLI ≥ 60 rather than imaging techniques or liver biopsy, which remain the gold standards for diagnosing hepatic steatosis and assessing fibrosis. Although FLI has been widely validated and is particularly advantageous in large-scale epidemiological studies for identifying individuals with probable hepatic steatosis [16], it does not provide information on the severity of steatosis or the stage of fibrosis [47]. Consequently, misclassification is possible, particularly in individuals with borderline FLI values or those with lean MASLD phenotypes, potentially leading to underestimation or overestimation of disease burden. Second, the cardiometabolic dysfunction burden was assessed only at baseline, which precluded the evaluation of longitudinal changes in metabolic risk profiles. As such, the number of risk factors may have varied during follow-up, potentially leading to misestimation of their true impact on MASLD prognosis. Future studies employing time-varying covariate models are warranted to address this limitation and reduce immortal time bias [48]. Third, due to the small number of individuals who failed to achieve metabolic control among those with only one or two cardiometabolic risk factors, stratified analyses of treatment response were conducted only in MASLD patients with all three risk factors. As a result, our findings regarding the prognostic impact of full, partial, or absent metabolic control were limited to this high-risk subgroup. Future studies with larger samples are warranted to assess whether similar treatment-response patterns exist in MASLD patients with fewer metabolic risk factors. Meanwhile, due to the lack of detailed treatment and follow-up data in NHANES, we were unable to evaluate heterogeneity in treatment response, which may confound the observed associations. Also, it is important to note that alcohol consumption in NHANES was assessed through self-reported questionnaires, which may lead to underestimation due to recall or social desirability bias. Previous studies have demonstrated the tendency of participants to under-report alcohol use in surveys [49], which may have influenced the diagnostic classification of MASLD in this study. Although the NHANES dataset provides a comprehensive representation of the U.S. population, results should be interpreted cautiously when extrapolating these findings to non-Western populations. Variations in genetic predispositions, dietary habits, healthcare access, and environmental factors may influence the burden of cardiometabolic dysfunction and its impact on MASLD prognosis [50]. Future studies involving diverse ethnic and geographic cohorts are needed to validate and extend our findings globally.

## 5. Conclusion

In this nationally representative, population-based cohort study, a higher burden of cardiometabolic dysfunction was significantly associated with increased mortality risk among individuals with MASLD. These findings highlight that cardiometabolic risk factors should not only be diagnostic components of MASLD but also function as key prognostic indicators. Patients harboring multiple cardiometabolic abnormalities, particularly those with diabetes mellitus and hypertension, may benefit from intensified clinical surveillance and targeted interventions to mitigate long-term mortality risk. Future longitudinal studies are warranted to validate these observations and to elucidate the relative prognostic weight of individual cardiometabolic components, thereby improving risk stratification and enabling personalized management strategies for the MASLD population.

## Supporting information

S1 Fig The association between the number of cardiometabolic risk factors and fatty liver index. CMRFs: cardiometabolic risk factors; data was displayed as median with 95%CI. Student-t test *P* < 0.05 indicates statistically significant. ^***^P < 0.001.
(PDF)

S2 Fig The Kaplan-Meier analysis (Log-rank test) to display the survival rate of obese participants with different types of cardiometabolic risk factors (n = 1). BP: blood pressure; Glu: blood glucose; HDL: High-density lipoprotein cholesterol; TG: Triglyceride. (Left) All-cause mortality; (Right) Cardiovascular mortality.
(PDF)

S3 Fig The Kaplan-Meier analysis (Log-rank test) to display the survival rate of obese participants with different types of cardiometabolic risk factors (n = 2). BP: blood pressure; Glu: blood glucose; HDL: High-density lipoprotein cholesterol; TG: Triglyceride. (Left) All-cause mortality; (Right) Cardiovascular mortality.
(PDF)

S4 Fig The Kaplan-Meier analysis (Log-rank test) to display the survival rate of obese participants with different types of cardiometabolic risk factors (n = 3). BP: blood pressure; Glu: blood glucose; HDL: High-density lipoprotein cholesterol; TG: Triglyceride. (Left) All-cause mortality; (Right) Cardiovascular mortality.
(PDF)

S1 Table The five cardiometabolic risk factors for the diagnosis of MASLD. (PDF)

S2 Table Mortality risk by metabolic control status in MASLD subgroup with three cardiometabolic risk factors. (PDF)

S3 Table Mortality risk by different metabolic risk factors in treated MASLD subgroup. (PDF)

S4 Table Sensitivity analysis of the association between the number of cardiometabolic risk factors and all-cause mortality in MASLD participants, stratified by FIB-4 defined intermediate-to-high advanced fibrosis risk. (PDF)

S5 Table Sensitivity analysis to check the stable association between the number of cardiometabolic risk factors and all-cause as well as cardiovascular mortality among participants with MASLD (Excluding participants who died within 48 months after the interview). (PDF)

S6 Table Sensitivity analysis to check the stable association between the number of cardiometabolic risk factors and all-cause as well as cardiovascular mortality among participants with MASLD. (Participants from the cycles between 1999–2008).
(PDF)

S7 Table Sensitivity analysis to check the stable association between the number of cardiometabolic risk factors and all-cause as well as cardiovascular mortality among participants with MASLD. (SLD defined by using FLI ≥ 30).
(PDF)
