# Supplementary figures and images for: Cardiometabolic dysfunction burden and mortality outcomes in metabolic dysfunction-associated steatotic liver disease
Source: PLoS One. 2025 Jul 3;20(7):e0327772. doi: 10.1371/journal.pone.0327772 (PMC12225798; doi:10.1371/journal.pone.0327772)

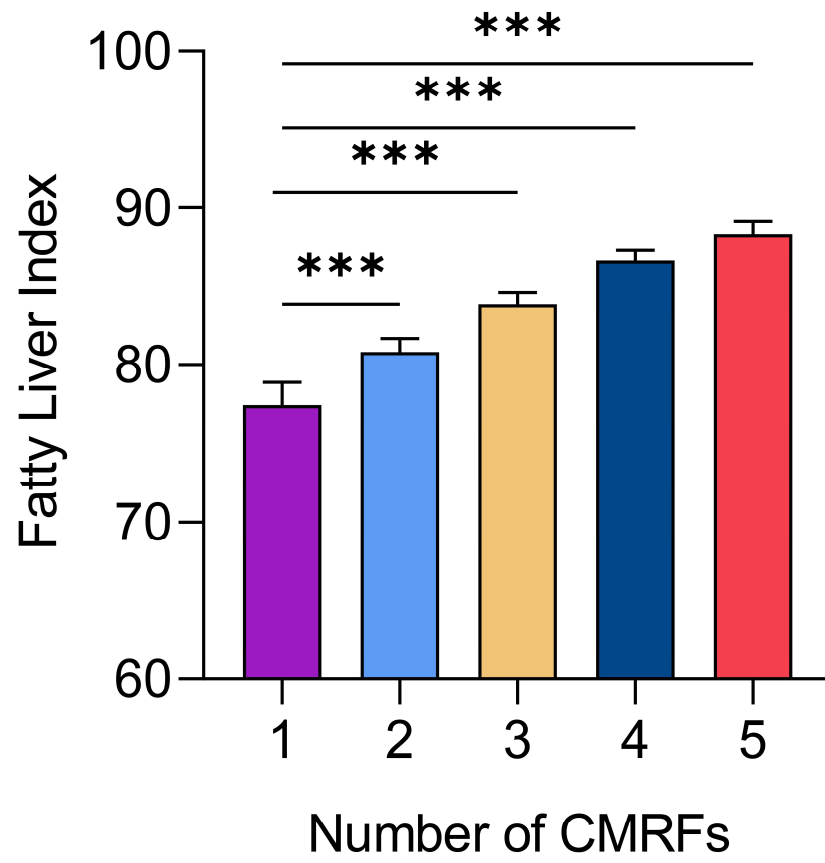

Supplement: S1 Fig — CMRFs: cardiometabolic risk factors; data was displayed as median with 95%CI. Student-t test P < 0.05 indicates statistically significant. ***P < 0.001. (PDF) [file pone.0327772.s001.pdf]

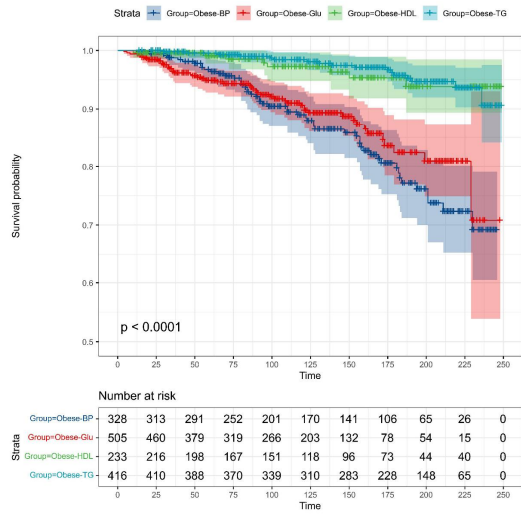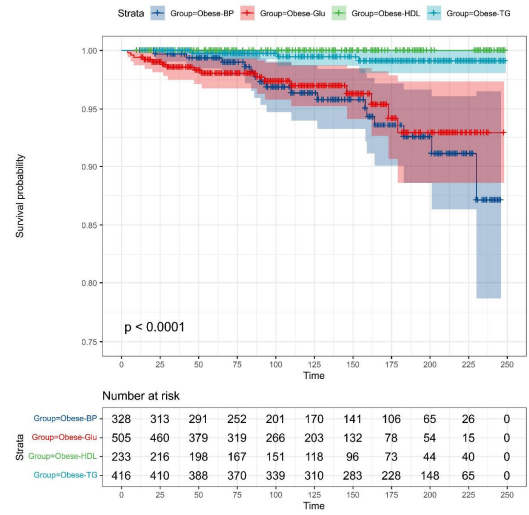

Supplement: S2 Fig — BP: blood pressure; Glu: blood glucose; HDL: High-density lipoprotein cholesterol; TG: Triglyceride. (Left) All-cause mortality; (Right) Cardiovascular mortality. (PDF) [file pone.0327772.s002.pdf]

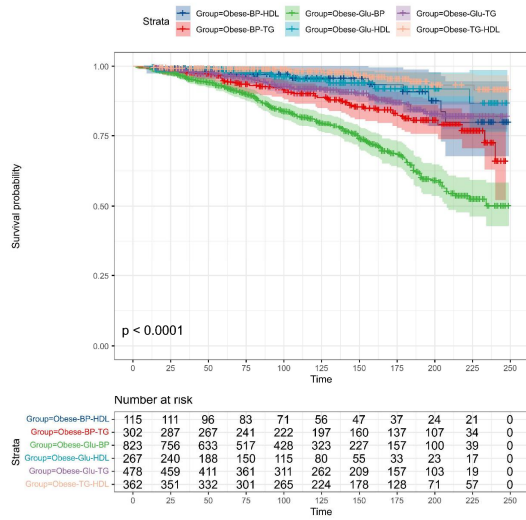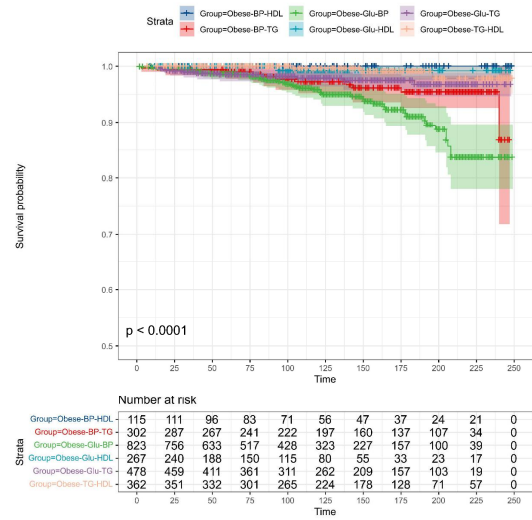

Supplement: S3 Fig — BP: blood pressure; Glu: blood glucose; HDL: High-density lipoprotein cholesterol; TG: Triglyceride. (Left) All-cause mortality; (Right) Cardiovascular mortality. (PDF) [file pone.0327772.s003.pdf]

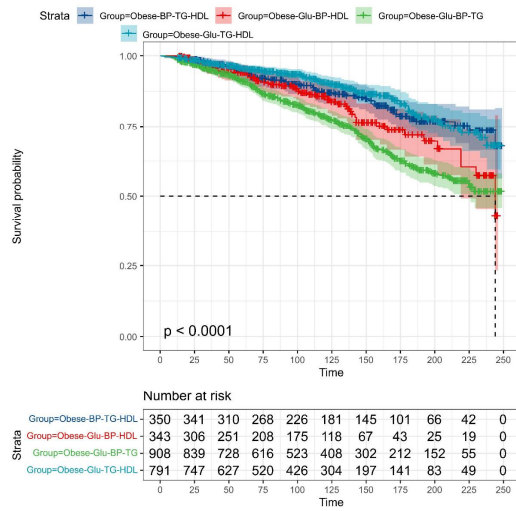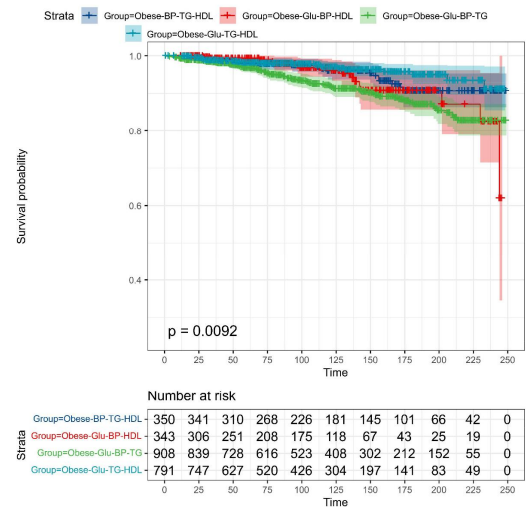

Supplement: S4 Fig — BP: blood pressure; Glu: blood glucose; HDL: High-density lipoprotein cholesterol; TG: Triglyceride. (Left) All-cause mortality; (Right) Cardiovascular mortality. (PDF) [file pone.0327772.s004.pdf]
