# Supplementary material for: Cardiometabolic dysfunction burden and mortality outcomes in metabolic dysfunction-associated steatotic liver disease
Source: PLoS One. 2025 Jul 3;20(7):e0327772. doi: 10.1371/journal.pone.0327772 (PMC12225798; doi:10.1371/journal.pone.0327772)
Supplement: S1 Table — (PDF) [file pone.0327772.s005.pdf]

S1 Table. The five cardiometabolic risk factors for the diagnosis of MASLD.

Types of abnormality	Met one of the listed criteria can be diagnosed as MASLD
**BMI/WC**	BMI ≥25 kg/m^2 or WC ≥94 cm for males and ≥80 cm for females
**Blood glucose**	FBG ≥100 mg/dl or 2-hour post-load glucose levels ≥140mg/dl or hemoglobin A1c ≥5.7% or DM or undergoing hypoglycemic therapy for DM
**Blood pressure**	Blood pressure ≥130/85 mmHg or undergoing antihypertensive drug treatment
**TG**	Fasting plasma TG ≥150 mg/dl or undergoing lipid-lowering treatment
**HDL-c**	Plasma HDL-cholesterol <40 mg/dl for males and <50 mg/dl for females

Abbreviation: MASLD: metabolic dysfunction-associated steatotic liver disease; BMI: body mass index; WC: waist circumference; FBG: fasting blood glucose; DM: diabetes mellitus; TG: triglyceride; HDL-c: high-density lipoprotein-cholesterol.
