# Supplementary material for: Cardiometabolic dysfunction burden and mortality outcomes in metabolic dysfunction-associated steatotic liver disease
Source: PLoS One. 2025 Jul 3;20(7):e0327772. doi: 10.1371/journal.pone.0327772 (PMC12225798; doi:10.1371/journal.pone.0327772)
Supplement: S2 Table — (PDF) [file pone.0327772.s006.pdf]

**S2 Table**. Mortality risk by metabolic control status in MASLD subgroup with three cardiometabolic risk factors.

| Groups | Model 1 | | Model 2 | | Model 3 | |
|---|---|---|---|---|---|---|
| | HR (95% CI) | *P* | HR (95% CI) | *P* | HR (95% CI) | *P* |
| All-cause mortality | | | | | | |
| Full control | Reference | | Reference | | Reference | |
| Partial control | 1.19(0.84-1.68) | 0.322 | 1.32(0.93-1.88) | 0.126 | 1.21(0.83-1.76) | 0.315 |
| Absent metabolic control | 1.66(1.00-2.74) | **0.048** | 1.83(1.10-3.04) | **0.020** | 2.20(1.26-3.86) | **0.006** |

Abbreviation: MASLD: metabolic dysfunction-associated steatotic liver disease; HR: hazard ratio; CI: confidence interval.

Note: Model 1: unadjusted model; Model 2: adjusted for age, sex, and race; Model 3: adjusted for age, sex, race, marital status, educational level, poverty income ratio, energy intakes, smoking status, alcohol use, CVD, CKD, cancer, AST, ALT, TBil, and TC. Bold value means statistically significant ($P < 0.05$) by using the Wald test.
