# Supplementary material for: Cardiometabolic dysfunction burden and mortality outcomes in metabolic dysfunction-associated steatotic liver disease
Source: PLoS One. 2025 Jul 3;20(7):e0327772. doi: 10.1371/journal.pone.0327772 (PMC12225798; doi:10.1371/journal.pone.0327772)
Supplement: S3 Table — (PDF) [file pone.0327772.s007.pdf]

S3 Table. Mortality risk by different metabolic risk factors in treated MASLD subgroup.

Groups	Model 1		Model 2		Model 3	
	HR (95% CI)	*P*	HR (95% CI)	*P*	HR (95% CI)	*P*
All-cause mortality						
One risk factor	Reference		Reference		Reference	
Two risk factors	1.47(1.05-2.05)	**0.026**	1.06(0.76-1.49)	0.731	1.09(0.78-1.53)	0.619
Three risk factors	2.20(1.61-3.01)	**<.001**	1.42(1.04-1.95)	**0.029**	1.42(1.03-1.95)	**0.032**

Abbreviation: MASLD: metabolic dysfunction-associated steatotic liver disease; HR: hazard ratio; CI: confidence interval.

Note: Model 1: unadjusted model; Model 2: adjusted for age, sex, and race; Model 3: adjusted for age, sex, race, marital status, educational level, poverty income ratio, energy intakes, smoking status, alcohol use, CVD, CKD, cancer, AST, ALT, TBil, and TC. Bold value means statistically significant ($P < 0.05$) by using the Wald test.
