# Supplementary material for: Cardiometabolic dysfunction burden and mortality outcomes in metabolic dysfunction-associated steatotic liver disease
Source: PLoS One. 2025 Jul 3;20(7):e0327772. doi: 10.1371/journal.pone.0327772 (PMC12225798; doi:10.1371/journal.pone.0327772)
Supplement: S4 Table — (PDF) [file pone.0327772.s008.pdf]

S4 Table. Sensitivity analysis of the association between the number of cardiometabolic risk factors and all-cause mortality in MASLD participants, stratified by FIB-4 defined intermediate-to-high advanced fibrosis risk.

Groups	Model 1		Model 2		Model 3	
	HR (95% CI)	*P*	HR (95% CI)	*P*	HR (95% CI)	*P*
FIB-4 <1.3						
1	Reference		Reference		Reference	
2	1.95(0.92-4.15)	0.083	1.97(0.93-4.20)	0.078	1.79(0.84-3.81)	0.131
3	4.56(2.23-9.30)	**<.001**	4.57(2.24-9.32)	**<.001**	3.64(1.78-7.45)	**<.001**
4	7.35(3.62-14.93)	**<.001**	7.35(3.62-14.94)	**<.001**	5.07(2.48-10.37)	**<.001**
5	12.81(6.31-25.99)	**<.001**	12.59(6.20-25.58)	**<.001**	7.09(3.46-14.51)	**<.001**
FIB-4 ≥1.3						
1	Reference		Reference		Reference	
2	3.73(1.51-9.21)	**0.004**	3.83(1.55-9.45)	**0.004**	2.90(1.17-7.16)	**0.021**
3	3.74(1.54-9.09)	**0.004**	4.07(1.67-9.89)	**0.002**	3.15(1.29-7.67)	**0.012**
4	4.04(1.67-9.79)	**0.002**	4.45(1.84-10.78)	**0.001**	3.36(1.39-8.16)	**0.007**
5	4.31(1.78-10.43)	**0.001**	4.71(1.95-11.41)	**0.001**	3.40(1.40-8.24)	**0.007**

Abbreviation: MASLD: metabolic dysfunction-associated steatotic liver disease; FIB-4: Fibrosis-4; HR: hazard ratio; CI: confidence interval.
Note: Model 1: unadjusted model; Model 2: adjusted for sex and race; Model 3: adjusted for sex, race, marital status, educational level, poverty income ratio, energy intakes, smoking status, alcohol use, CVD, CKD, cancer, TBil, and TC. Bold value means statistically significant ($P < 0.05$) by using the Wald test.
