# Supplementary material for: Cardiometabolic dysfunction burden and mortality outcomes in metabolic dysfunction-associated steatotic liver disease
Source: PLoS One. 2025 Jul 3;20(7):e0327772. doi: 10.1371/journal.pone.0327772 (PMC12225798; doi:10.1371/journal.pone.0327772)
Supplement: S7 Table — (SLD defined by using FLI ≥ 30). (PDF) [file pone.0327772.s011.pdf]

S7 Table. Sensitivity analysis to check the stable association between the number of cardiometabolic risk factors and all-cause as well as cardiovascular mortality among participants with MASLD. (SLD defined by using FLI ≥30).

Groups	Model 1		Model 2		Model 3	
	HR (95% CI)	*P*	HR (95% CI)	*P*	HR (95% CI)	*P*
All-cause mortality						
1	Reference		Reference		Reference	
2	2.52(1.85-3.43)	**<.001**	1.84(1.35-2.51)	**<.001**	1.78(1.30-2.42)	**<.001**
3	4.88(3.63-6.55)	**<.001**	2.44(1.81-3.29)	**<.001**	2.24(1.66-3.03)	**<.001**
4	7.40(5.52-9.93)	**<.001**	2.81(2.09-3.79)	**<.001**	2.39(1.77-3.23)	**<.001**
5	8.56(6.29-11.64)	**<.001**	3.14(2.29-4.29)	**<.001**	2.51(1.83-3.44)	**<.001**
Cardiovascular mortality						
1	Reference		Reference		Reference	
2	2.60(1.38-4.91)	**0.003**	1.90(1.01-3.60)	**0.048**	1.87(0.99-3.54)	0.055
3	5.39(2.93-9.91)	**<.001**	2.65(1.43-4.90)	**0.002**	2.49(1.34-4.61)	**0.004**
4	9.45(5.17-17.27)	**<.001**	3.57(1.93-6.58)	**<.001**	3.06(1.65-5.67)	**<.001**
5	11.38(6.09-21.26)	**<.001**	4.26(2.25-8.05)	**<.001**	3.41(1.79-6.47)	**<.001**

Abbreviation: MASLD: metabolic dysfunction-associated steatotic liver disease; FLI: fatty liver index; HR: hazard ratio; CI: confidence interval.

Note: Model 1: unadjusted model; Model 2: adjusted for age, sex, and race; Model 3: adjusted for age, sex, race, marital status, educational level, poverty income ratio, energy intakes, smoking status, alcohol use, CVD, CKD, cancer, AST, ALT, TBil, and TC. Bold value means statistically significant (P < 0.05) by using the Wald test.
